# *TcG2/TcG4* DNA Vaccine Induces Th1 Immunity Against Acute *Trypanosoma cruzi* Infection: Adjuvant and Antigenic Effects of Heterologous *T. rangeli* Booster Immunization

**DOI:** 10.3389/fimmu.2019.01456

**Published:** 2019-06-26

**Authors:** Shivali Gupta, Berenice Salgado-Jiménez, Nandadeva Lokugamage, Juan Carlos Vázquez-Chagoyán, Nisha Jain Garg

**Affiliations:** ^1^Department of Microbiology and Immunology, University of Texas Medical Branch, Galveston, TX, United States; ^2^Facultad de Medicina Veterinaria y Zootecnia, Centro de Investigación y Estudios Avanzados en Salud Animal, Universidad Autónoma del Estado de México, Toluca, Mexico

**Keywords:** *Trypanosoma cruzi*, Chagas disease, recombinant DNA vaccine, *T. rangeli*, immune efficacy

## Abstract

**Background:** Chagas cardiomyopathy is caused by *Trypanosoma cruzi* (*Tc*). Two antigenic candidates, TcG2 and TcG4, are recognized by antibodies in naturally infected dogs and humans; and these vaccine candidates provided protection from *Tc* infection in mice and dogs. *Trypanosoma rangeli* (*Tr*) is non-pathogenic to mammals and shown to elicit cross-reactive anti-*Tc* antibodies. In this study, we investigated if fixed *Tr* (fTr) can further enhance the efficacy of the *TcG2/TcG4* DNA vaccine.

**Methods and Results:** C57BL/6 mice were immunized with *TcG2/TcG4* DNA vaccine and fTr (delivered as an adjuvant or in prime-boost approach), and challenged with *Tc*. Serology studies showed that fTr (±quil-A) elicited *Tc*- and *Tr-*reactive IgGs that otherwise were not stimulated by *TcG2/TcG4* vaccine only, and quil-A had suppressive effects on fTr-induced IgGs. After challenge infection, *TcG2/TcG4*-vaccinated mice exhibited potent expansion of antigen- and *Tc*-specific IgGs that were not boosted by fTr±quil-A. Flow cytometry analysis showed that *TcG2/TcG4*-induced dendritic cells (DC) and macrophages (Mφ) responded to challenge infection by expression of markers of antigen uptake, processing, and presentation, and production of pro-inflammatory cytokines. *TcG2/TcG4*-induced CD4^+^T cells acquired Th1 phenotype and expressed markers that orchestrate adaptive immunity. A fraction of vaccine-induced CD4^+^T cells exhibited iTreg phenotype responsible for aversion of self-injurious immune responses. Further, *TcG2/TcG4*-vaccinated mice exhibited potent expansion of poly-functional CD8^+^T cells with TNF-α/IFN-γ production and cytolytic phenotype post-infection. Subsequently, tissue parasites and pathology were hardly detectable in *TcG2/TcG4*-vaccinated/infected mice. Inclusion of fTr±quil-A had no clear additive effects in improving the *Tc*-specific adaptive immunity and parasite control than was noted in mice vaccinated with *TcG2/TcG4* alone. Non-vaccinated mice lacked sufficient activation of Th1 CD4^+^/CD8^+^T cells, and exhibited >10-fold higher levels of tissue parasite burden than was noted in vaccinated/infected mice.

**Conclusion:** *TcG2/TcG4* vaccine elicits highly effective immunity, and inclusion of fTr is not required to improve the efficacy of DNA vaccine against acute *Tc* infection in mice.

## Introduction

Chagas cardiomyopathy, caused by *Trypanosoma cruzi*, is a major health concern in Latin America and it is an emerging disease in the United States, Europe, Japan, and other countries (1). The majority of individuals exposed to *T. cruzi* remain seropositive for their life. In ~30% of the infected individuals, clinical symptoms progress from cardiac hypertrophic remodeling (i.e., wall thickening) to dilated cardiomyopathy, and ultimately result in cardiac arrest and death (2).

The sequencing of *T. cruzi* genome (3) and the development of approaches to produce recombinant proteins at low cost have made it feasible to produce, deliver, and test the efficacy of a variety of recombinant *T. cruzi* antigens as potential vaccine candidates in experimental models of infection and disease. We have screened several candidate antigens, and selected TcG1, TcG2, and TcG4 for further development as potential vaccine candidates. These antigens are phylogenetically conserved in clinically important *T*. *cruzi* strains, expressed in infective and intracellular stages of the parasite, and recognized by parasite-specific cellular and humoral immune responses in multiple *T*. *cruzi*-infected hosts (4–7). Further, we showed that prophylactic immunization with TcG1, TcG2, and TcG4 based subunit vaccine(s) elicited parasite-specific lytic antibodies and cytolytic T cell responses, and Th1 cytokines in mice and dogs (8–12). Recent studies have tested several other antigenic candidates as vaccine for their prophylactic and therapeutic efficacy against Chagas disease. Results of these vaccines are encouraging and summarized in recent reviews (13–15). However, till to date none of the anti-*T*. *cruzi* vaccines have produced sterile immunity in any of the experimental animal models.

The use of heterologous DNA-prime/inactivated microorganism-boost vaccine (13) has been previously reported with promising results. *T*. *rangeli* (*Tr*) exhibits significant homology (>60%) with *T*. *cruzi* proteome (15, 16), but *Tr* is not pathogenic for mammals (17, 18), and, therefore, it can be cultivated in large batches in clean, biosafety level one laboratory facilities. Studies in mice and dogs have suggested that immunization with fixed, lysed, or fractionated *Tr* can provide a degree of protection from *T*. *cruzi* infection and histopathological lesions produced during Chagas disease (19–21). When used in combination with a subunit vaccine, booster immunization with glutaraldehyde fixed *T*. *rangeli* (fTr) epimastigotes appeared to lower the blood parasitemia and tissue parasite foci in dogs (12). However, the immunological mechanisms that might be elicited by fTr to provide protection against *T. cruzi* infection are not known.

In this study, we aimed to determine if fTr enhances the efficacy of the DNA vaccine against *T. cruzi*. For this, we immunized mice with *TcG2/TcG4* DNA and fTr (individually or in combination, or in a prime-boost approach), and then challenged with *T. cruzi*. We analyzed the reactivity of the vaccine-induced antibody responses against *T. cruzi* and *T. rangeli*, and profiled the activation of antigen presenting cells (APC), i.e., dendritic cells (DC) and macrophages (Mφ), and CD4^+^ and CD8^+^ T cells after vaccination and challenge infection. We also evaluated the efficacy of vaccine compositions in controlling parasite dissemination in tissues and examined histopathology of the heart and skeletal muscles, focusing on the efficacy of vaccination protocol in reducing the tissue injury by acute *T. cruzi* infection.

## Materials and Methods

### Ethics Statement

All animal experiments were conducted following the National Institutes of Health guidelines for housing and care of laboratory animals and in accordance with protocols approved by the Institutional Animal Care and Use Committee (protocol number 08-05-029) at The University of Texas Medical Branch at Galveston.

All experiments were conducted in ABSL2/BSL2-approved laboratory and all personnel have received appropriate ABSL2/BSL2 training.

### Vaccine Composition, Immunization, and Challenge Infection

The cDNAs for TcG2 and TcG4 (Genbank: AY727915 and AY727917, respectively) were cloned in pCDNA3.1 eukaryotic expression plasmid (10). Recombinant plasmids were transformed into *E*. *coli* DH5-alpha-competent cells, grown in Luria-Bertani broth containing 100 μg/ml ampicillin, and purified by anion exchange chromatography by using a Qiagen Endofree maxi prep kit (Qiagen, Chatsworth, CA) (4). Purified plasmids were used at a concentration of 25 μg each plasmid per vaccination.

*T*. *rangeli* (*Tr*, Guatemala strain) epimastigote cultures were propagated in LIT media (12). *T*r epimastigotes (1 × 10^9^/ml) were fixed with 0.1% glutaraldehyde solution (Sigma-Aldrich), washed three times with 1X cold Phosphate Buffered Saline (PBS). Fixed *Tr* (fTr) lysate (1 × 10^8^ *Tr* equivalent in 100 μl PBS) was used with or without 5 μg quil A (QA) for vaccination (12).

C57BL/6 female mice (6-week-old) were obtained from Harlan Labs (Indianapolis, IN). To assess if fTr and Quil A (QA, saponin) adjuvant the DNA vaccine-induced responses, mice were vaccinated in following groups: (1) DNA vaccine only, two doses; (2) DNA vaccine + fTr, two doses; (3) DNA vaccine + QA, two doses; (4) DNA vaccine + fTr + QA, two doses. To determine if fTr boosts the DNA vaccine-induced immune responses, mice were vaccinated with DNA vaccine followed by fTr (gp5) or fTr+QA (gp6). Each dose of DNA vaccine was constituted of 25-μg of each plasmid (*pCDNA3.TcG2* and *pCDNA3.TcG4*) and delivered in 100 μl PBS by intramuscular (im) injection in the hind thighs. When used, fTr (1 × 10^8^ *Tr* in 100 μl PBS) was delivered by subcutaneous (sc) injection. When added, vaccine was emulsified with 5 μg QA per dose per mouse. Non-vaccinated (N) mice were used as controls. Prime and booster doses of vaccine were given at 21-day intervals. Immunized and control mice were euthanized at 21 days' post-vaccination (pv), and sera, splenocytes, and lymph node (LN) cells were collected to evaluate the vaccine-induced immunological responses.

**Figure 1:**
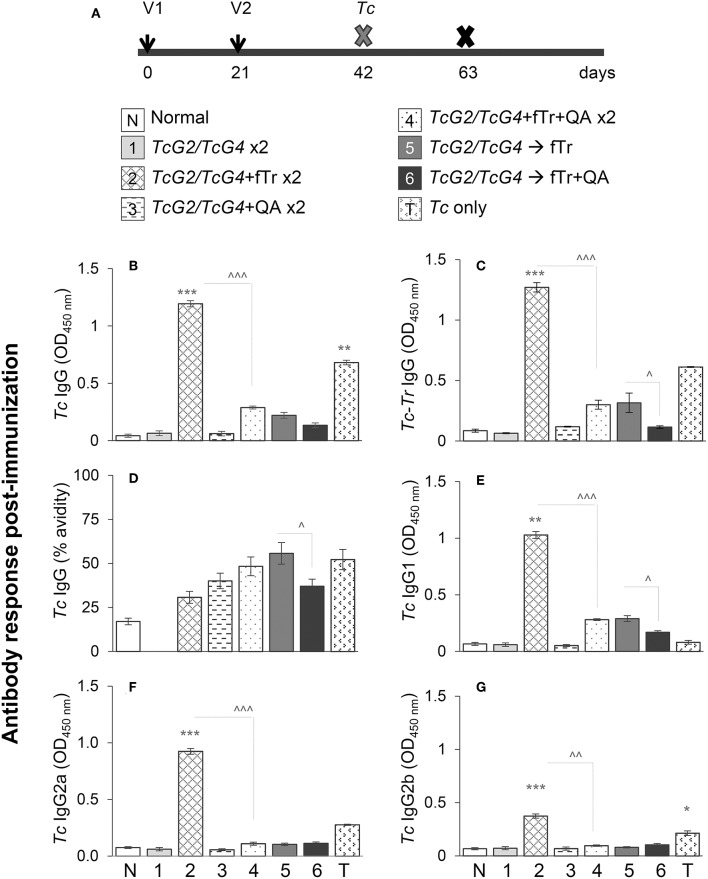
*T. cruzi*-specific antibody response in vaccinated mice. **(A)** Schematic of experimental plan is shown. C57BL/6 female mice were immunized with six different compositions of vaccines as described in Materials and Methods. Mice were immunized with dose 1 at day 0, dose 2 at day 21, and on day 42 euthanized to examine the vaccine-induced responses or challenged with *Tc* and then euthanized at day 63 to study the significance of vaccine in providing protection from infection. Vaccines (per dose) were constituted with 25-μg each of pCDNA3.*TcG2* and pCDNA3.*TcG4* plasmid DNA (intramuscular) and/or 1 × 10^8^ *T. rangeli* fixed with 0.1% glutaraldehyde (fTr) with or without 5-μg Quil A (QA, subcutaneous). **(B–G)** *T. cruzi*-specific IgG **(B)**, IgG1 **(E)**, IgG2a **(F)**, and IgG2b **(G)** antibodies in sera samples were measured by an ELISA. The vaccine-induced IgGs specificity for *T. cruzi* was determined after pre-adsorbing the sera samples with *T. rangeli* soluble lysate ***(C)*** and avidity of vaccine induced IgGs was calculated as [O.D. with urea/O.D. without urea treatment] × 100 **(D)**. Sera samples from non-vaccinated or infected mice were used as negative and positive controls, respectively. Data (mean ± SD) are representative of two independent experiments (*n* = 4 mice per group per experiment, duplicate observations per sample), and significance is presented as * (none vs. vaccinated) or ^∧^ (comparison among vaccinated groups). The *p-*values of <0.05, <0.01, and *p* < 0.001 are annotated with one, two, and three symbols, respectively.

For challenge infection, *T*. *cruzi* (*Tc*) trypomastigotes (Sylvio X10/4 strain) were maintained and propagated by continuous *in vitro* passage in C2C12 cells. We included gp1, gp5, and gp6 for challenge studies because mice in these groups exhibited maximal vaccine-induced T cell immunity. Mice were immunized as above, challenged with *Tc* (10,000 trypomastigotes/mouse, intraperitoneal) at 21 days after the 2nd vaccine dose, and euthanized at 21 days' post-infection (pi). Non-vaccinated mice infected with *Tc* (T) and euthanized at similar time-points were used as controls. The vaccination and challenge infection scheme is presented in Figure 1A.

### Serology

The cDNAs for *TcG2* and *TcG4* were cloned in-frame with a C-terminal His-tag into a pET-22b plasmid (Novagen, Gibbstown, NJ). Plasmids were transformed in *BL21* (DE3) pLysS-competent cells, and recombinant proteins were purified by using the poly-histidine fusion, peptide-metal chelation chromatography system (8, 11). *Tc* trypomastigotes and *Tr* epimastigotes (2 × 10^7^ each) were sonicated in 1 mL of 50 mM carbonate-bicarbonate buffer (pH 9.6), and centrifuged for 10 min at 10,000 × *g* at 4°C. The supernatants of *Tc* lysate (TcL) and *Tr* lysate (TrL) were used as soluble antigens for serology.

Serum immunoglobulin G (IgG) response was monitored by an enzyme linked immunosorbent assay (ELISA). Briefly, 96-well flat-bottom plates (Falcon, Becton Dickinson, Oxnard, CA) were coated for 2 h with TcL or TrL (5 × 10^5^ protozoans' equivalent/well) or recombinant TcG2 and TcG4 proteins (10-μg/ml). Plates were blocked for 1 h with 5% non-fat dry milk (NFDM, Bio-Rad) in PBS-0.01% Tween 20 (PBST). Plates were washed and incubated for 2 h with sera from vaccinated, vaccinated/infected and control mice (1:100–1:1,000 dilutions in 0.5% NFDM, 100 μl/well). To detect the total IgG levels, plates were then directly incubated for 30 min with goat anti-mouse IgG–horseradish peroxidase (HRP) conjugate. To detect IgG subtypes, plates were sequentially incubated for 2 h with biotin-conjugated goat anti-mouse Ig subtypes (IgG1, IgG2a, IgG2b, or IgG2c), and for 30 min with streptavidin-HRP conjugate. All incubations were carried at 37°C, and plates were washed at each step with PBST and PBS. All antibodies were purchased from Southern Biotech, and used at 1: 5,000 dilutions in PBST-0.5% NFDM. Color was developed by incubation with 100 μl/well Sure Blue TMB substrate (Kirkegaard & Perry Labs) and recorded at 450 nm by using a SpectraMax M5 microplate reader (Molecular Devices). In some experiments, sera samples were pre-absorbed with TcL or TrL for 1 h before use in an ELISA assay (8, 16).

To examine the antigen-avidity index, plates were coated with TcL as above, blocked with 5% NFDM, and sequentially incubated with sera samples (1: 100 dilutions in 1% NFDM) for 1 h, 6 M urea for 30 min, and HRP-conjugated secondary antibody for 30 min. A colorimetric reaction was performed as above (% Avidity: [O.D. with urea/O.D. without urea treatment] × 100) (17).

### Flow Cytometry Characterization of Immune Responses

Single-cell suspensions of spleen or lymph nodes (LN) from vaccinated, vaccinated/infected, non-vaccinated/infected, and non-vaccinated/non-infected mice were prepared by standard methods. Splenic and LN cell counts in vaccinated and control mice are presented in Table S1. Splenocytes or LN cells (10^5^ cells/100 μl color-free RPMI) were used immediately for flow cytometry. In some experiments, splenocytes were *in vitro* stimulated with TcL (25-μg/ml) at 37°C, 5% CO_2_ for 48 h. The un-stimulated and *in vitro-* stimulated cells were washed in staining buffer (2% BSA/0.02% sodium azide in PBS) and incubated for 15 min with Fc Block (anti-CD16/CD32; BD Pharmingen). Cells were then incubated for 30 min at 4°C in dark with the fluorochrome-conjugated antibodies, washed twice in PBS, fixed in 2% paraformaldehyde, and washed again. Fluorescent cells were visualized by using a FACS-Calibur Cell Analyzer (BD Biosciences), acquiring >20,000 events in a live gate, and further analyzed by using FlowJo software (version 7.6.5, Tree-Star, San Carlo, CA) (22).

For the measurement of intracellular cytokines and immune cell activation markers, splenocytes were stimulated as above except that brefeldin A (10-μg/ml, Sigma) or monensin (5 μg/ml) (BD Pharmingen) was added for the final 6 h of culture to block protein secretion. Cells were then labeled with antibodies for surface markers, fixed with 2% paraformaldehyde, re-suspended in 100-μl permeabilization buffer (0.1% saponin/1% FBS in PBS), and utilized for intracellular staining of cytokines or specific markers. Samples were analyzed by flow cytometry as above. In all experiments, unstained cells and cells stained with isotype-matched IgGs and FMO were used as controls (13, 22).

All antibodies used for flow cytometry in this study are listed in Table S2. Briefly, to profile the antigen presenting cells, splenic and LN cells of all vaccinated and control groups before and challenge infection were first gated for Ly6G^lo^CD11c^+^ dendritic cells (DC) and Ly6G^lo^Ly6C^+^CD11b^+^ monocytes/macrophages (Mφ). Each of the cell populations were further analyzed by flow cytometry for the expression of CD209 (DC-SIGN) C-type lectin receptor that recognizes PAMPs and activates phagocytosis and/or antigen presentation, CD205 that is involved in internalization, processing and presentation of antigens, MHCI/MHCII molecules that bind to peptide fragments from pathogens and present to the T cells, and CD80 that is the ligand for CD28 and CTLA4 and provides co-stimulatory signal for adaptive T cell response. The splenic CD11b^+^ Mφ cell subsets were also analyzed for intracellular expression of markers of classical/proinflammatory (IL-1β^hi^ and TNF-α^hi^) and alternative/immunomodulatory (CD200^+^, CD206^+^) functional phenotype after *in vitro* stimulation with TcL (25-μg/mL) for 48 h (18).

To evaluate the T cell profile, splenocytes from vaccinated, vaccinated/infected, and control groups were *in vitro* stimulated with TcL (25-μg/mL) for 48 h. Splenic CD3^+^ lymphocytes were gated for CD4^+^ and CD8^+^ T cells. Each of the T cell subsets were further analyzed for the expression of CD69 the early activation marker of lymphocyte proliferation, CD11a (ITGAL) that along with CD18 forms lymphocyte function-associated antigen 1 (LFA-1) and binds with ICAMs to facilitate intercellular adhesion of leukocytes, CD95 (Fas) / CD95L (FasL) involved in immune elimination of infected cells, CD44 and CD62L molecules to distinguish naïve (CD62L^hi^CD44^lo^), effector (CD62L^lo^CD44^hi^), and central memory (CD62L^hi^CD44^hi^) phenotypes, and intracellular production of Th1 cytokines (IFN-γ, TNF-α). The CD4^+^T cells were also examined for the surface expression of CD25 and FoxP3 to profile the splenic T regulatory cells. The CD8^+^T cells were also analyzed for the intracellular expression of perforin and CD107a that are markers of cytolytic activity (10, 11).

### Histology and Tissue Parasite Burden

For histological studies, heart and skeletal muscle tissue sections from vaccinated/infected and non-vaccinated/infected mice were fixed in 10% buffered formalin for 24 h, dehydrated in absolute alcohol, cleared in xylene, and embedded in paraffin. Paraffin-embedded 5-micron tissue-sections were stained with hematoxylin and eosin (H&E) and evaluated by light microscopy. Heart and skeletal muscle tissue slides (three mice per group, at least two slides per tissue, 10 microscopic fields per slide) were analyzed by light microscopy, and the presence of inflammatory cells was scored as (0)–absent/none, (1)–focal or mild with ≤ 1 foci, (2)–moderate with ≥2 inflammatory foci, (3)–extensive with generalized coalescing of inflammatory foci or disseminated inflammation (4)–severe with diffused inflammation, interstitial edema, and loss of tissue integrity (23).

To examine the parasite burden, heart and skeletal muscles tissues (10 mg) from vaccinated/infected and non-vaccinated/infected mice were subjected to proteinase K lysis, and total DNA was purified by phenol/chloroform extraction and ethanol precipitation. A real-time quantitative PCR was performed on an iCycler thermal cycler with SYBR Green Supermix (Bio-Rad), 50 ng of total DNA, and oligonucleotides specific for *Tc*18SrDNA (forward, 5′- TTTTGGGCAACAGCAGGTCT-3′; reverse, 5′- CTGCGCCTACGAGACATTCC-3′; amplicon size: 199 bp) and murine *GAPDH* (forward, 5′-AACTTTGGCATTGTGGAAGG-3′; reverse, 5′-ACACATTGGGGGTAGGAACA-3′; amplicon size: 223 bp). The threshold cycle (C_*T*_) values for *Tc*18SrDNA were normalized to *GAPDH* reference cDNA. The relative parasite burden (i.e., *Tc18SrDNA* level) was calculated by following the 2^−ΔΔ*Ct*^, where Δ*Ct* represents the *CT* (sample)—*Ct* (*GAPDH*) and ΔΔCt represents ΔCt (sample)—ΔCt (no treatment control).

### Statistical Analysis

Data are expressed as mean ± SD (*n* = 4/group/experiment, minimum of duplicate observations per experiment). Data were analyzed by the Student's *t*-test (comparison of 2 groups) and 1-way analysis of variance (ANOVA) with Tukey's *post-hoc* test (comparison of multiple groups) by using an SPSS (version 14.0, SPSS Inc, Chicago, Illinois) or Graph Pad InStat ver.3 software. Significance is presented as ^*^ (vaccinated vs. non-vaccinated or vaccinated/infected vs. non-vaccinated/infected) and ^∧^ (comparison of vaccinated groups) and *p*-values of <0.05, <0.01, and 0.001 are annotated by one, two, or three symbols, respectively.

## Results

### Elicitation of Antibody Response and Immune Cell Expansion in Mice Immunized With DNA Vaccine (± fTr)

We first monitored if fTr, delivered as an adjuvant or as an antigen in prime-boost approach, modulates the *TcG2/TcG4* DNA vaccine—induced antibody response in mice. For this, sera samples were obtained at 21 days after the 2nd vaccine dose, and analyzed by an ELISA. Mice immunized/boosted with *TcG2/TcG4* (±QA, gp1, and gp3) exhibited no detectable levels of *Tc*-specific antibodies (Figures 1B,C). In comparison, all other vaccinated groups of mice that received fTr (±QA) as adjuvant or booster exhibited detectable, though variable, levels of *Tc*-specific IgGs with 25–51% avidity for *Tc* antigens (Figures 1B,C). Including fTr as an adjuvant with *TcG2/TcG4* vaccine (gp2) elicited maximal levels of *Tc*-specific IgGs (^*^p < 0.001) and addition of QA suppressed the fTr-induced IgG (compare gp2 vs. gp4, ^∧^*p* < 0.001) (Figures 1B,C). Interestingly, avidity of fTr induced antibodies for *Tc* antigens was minimal among all vaccinated groups (compare gp2 with other groups, Figure 1D). Likewise, *TcG2/TcG4* vaccine adjuvanted with fTr also resulted in maximal levels of *Tc*-specific IgG sub-types (IgG1, IgG2a, and IgG2b) (gp2 vs. all other groups, ^*^p < 0.01) while co-delivery of QA had a suppressive effect on *Tc*-specific IgG subtypes stimulated by *TcG2/TcG4* vaccine adjuvanted with fTr (^∧^*p* < 0.001, compare gp2 and gp4) or boosted with fTr (^∧^*p* < 0.05, compare gp5 and gp6) (Figures 1E–G).

*Tr*-specific IgGs constituted of IgG1, IgG2a, and IgG2b subtypes were also detected in mice that received fTr as an adjuvant with *TcG2/TcG4* DNA or as a booster vaccine, and maximal *Tr*-specific antibodies were measured in sera of mice given fTr as an adjuvant with *TcG2/TcG4* DNA vaccine (group 2 vs. control, ^*^p < 0.001, Figures S1A–D). Low levels of *Tr*-reactive IgGs were also observed in mice infected with *T. cruzi* (Figure S1B). Normal mice and mice injected with vector only exhibited no *Tc*- and *Tr*-specific antibody response. Together, the results presented in Figure 1 and Figure S1 suggest that (a) *TcG2/TcG4* DNA vaccine induced low levels of *Tc*- and antigen-specific antibodies, (b) co-delivery of fTr as an adjuvant elicited maximal levels of *Tc*- as well as *Tr*-reactive IgGs with minimal avidity for *Tc* antigens, and (c) addition of QA with fTr had an overall suppressive effect on antibody responses induced in vaccinated mice.

We evaluated the effect of vaccine compositions on the expansion of splenic and lymph node (LN) cells in mice. With respect to splenic cells, the overall frequency of splenocytes (range: 80–193-million cells) was increased by 62–141% in vaccinated (vs. control) mice (Table S1). Mice that received booster dose of *TcG2/TcG4*, fTr, or fTr+QA exhibited maximal expansion of splenocytes post-vaccination, while mice receiving TcG2/TcG4 adjuvanted with fTr, QA or fTr+QA exhibited minimal expansion of splenic immune cells (gp1, gp5, gp6, Table S1). With respect to lymph nodes, we noted up to 52% expansion of LN cells (range: 144-220-million cells) in vaccinated mice, and maximal expansion was noted in mice immunized with TcG2/TcG4 and boosted with fTr or fTr+QA (gp5 and gp6, Table S1). Based on the observations that fTr, QA, and fTr+QA adjuvants (gp2, gp3, gp4) did not enhance the TcG2/TcG4-induced expansion of splenic and LN cells (Table S1) as well as high avidity antibody responses (Figure 1), we excluded the gp2, gp3, and gp4 from further studies.

### Expansion of DNA Vaccine (± fTr)–Induced Antibody Response Upon Challenge Infection

**Figure 2:**
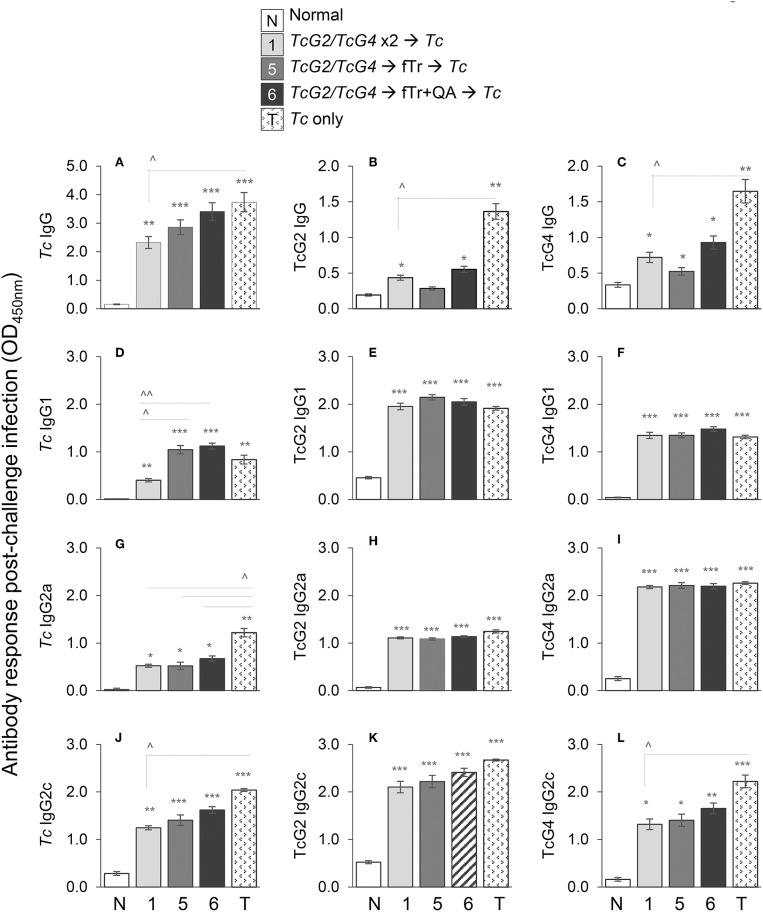
Antibody response in vaccinated mice after challenge infection. C57BL/6 female mice were immunized with subunit vaccine (pCDNA3.1 encoding TcG2 and TcG4) at day 0, boosted with subunit vaccine, fTr, or fTr+QA at day 21, and challenged with *T. cruzi* (10,000 parasites per mouse, intraperitoneal) at day 42. Mice were euthanized at day 63 (i.e., 21 days' post challenge infection). Sera samples were analyzed by an ELISA to evaluate the IgG **(A–C)**, IgG1 **(D–F)**, IgG2a **(G–I)**, and IgG2c **(J–L)** levels captured with *T. cruzi* lysate **(A,D,G,J)** and recombinant TcG2 **(B,E,H,K)**, and TcG4 **(C,F,I,L)** antigens. Sera samples from normal and infected mice were used as controls. Data (mean ± SD) are representative of duplicate observations per sample (*n* = 4 mice per group per experiment), and significance is annotated as * (none vs. vaccinated or infected vs. vaccinated/infected) and ^∧^ (comparison of vaccinated groups) (*,^∧^*p* < 0.05, **,^∧^^∧^*p* < 0.01, and ***p < 0.001).

To examine if vaccine-induced IgGs expanded in response to infection, mice were challenged with *T. cruzi* at 21 days' pv, and sera samples were obtained at 21 days' pi (corresponds to acute parasitemia phase). All mice challenged with *Tc*, irrespective of the vaccination regimen, exhibited significant levels of *Tc*-, TcG2-, and TcG4-specific IgGs (vs. normal controls, ^*^p < 0.05, Figures 2A–C). Booster immunization with fTr (±QA) slightly enhanced the hosts' capacity to respond to challenge infection with expansion of *Tc*-specific antibodies than was observed in *TcG2/TcG4*-immunized/infected mice (Figure 2A); however, booster immunization with fTr (±QA) did not enhance the TcG2- and TcG4-specific IgGs in DNA vaccinated mice (Figures 2B,C, compare gp5 and gp6 with gp1). The maximal levels of *Tc*- and antigen-specific IgGs were detected in infected mice that did not receive any vaccine (vs. vaccinated/infected, ^∧^*p* < 0.05, Figures 2A,C). We also monitored the *Tc* and antigen-specific antibody sub-types in immunized mice post challenge infection. Overall, all mice responded to challenge infection with a significant increase in *Tc*- *TcG2*- and *TcG4*-specific IgG1, IgG2a, and IgG2c antibody subtypes (vs. normal controls, ^*^p < 0.01, Figures 2D–L). Maximal levels of *Tc*- and TcG2/TcG4-specific antibody subtypes (IgG2a+IgG2c > IgG1) were detected in non-vaccinated/infected mice (vs. normal controls, ^*^p < 0.001, Figures 2D–L). Mice immunized with *TcG2/TcG4* and boosted with *TcG2/TcG4* also exhibited a predominance of *Tc* and antigen-specific IgG2a/2c (vs. IgG1) antibody sub-types post-challenge infection (compare Figures 2G–L with Figures 2D–F). Further, booster immunization with fTr (±QA) slightly, but non-significantly, enhanced the *Tc*- and antigen-specific antibody subtypes post-challenge infection than was observed in *TcG2/TcG4*-immunized/infected mice (Figures 2D–L). Together the results presented in Figure 2 suggest that (a) challenge infection elicits a predominance of antigen- and parasite-specific IgG2a/2c antibodies that are not enhanced by pre-immunization with DNA vaccine; and (b) fTr (±QA) did not improve the extent of antigen- and parasite-specific IgGs in *TcG2/TcG4*-vaccinated mice.

### DC Activation Profile in Vaccinated Mice Before and After Challenge Infection With *Tc*

**Figure 3:**
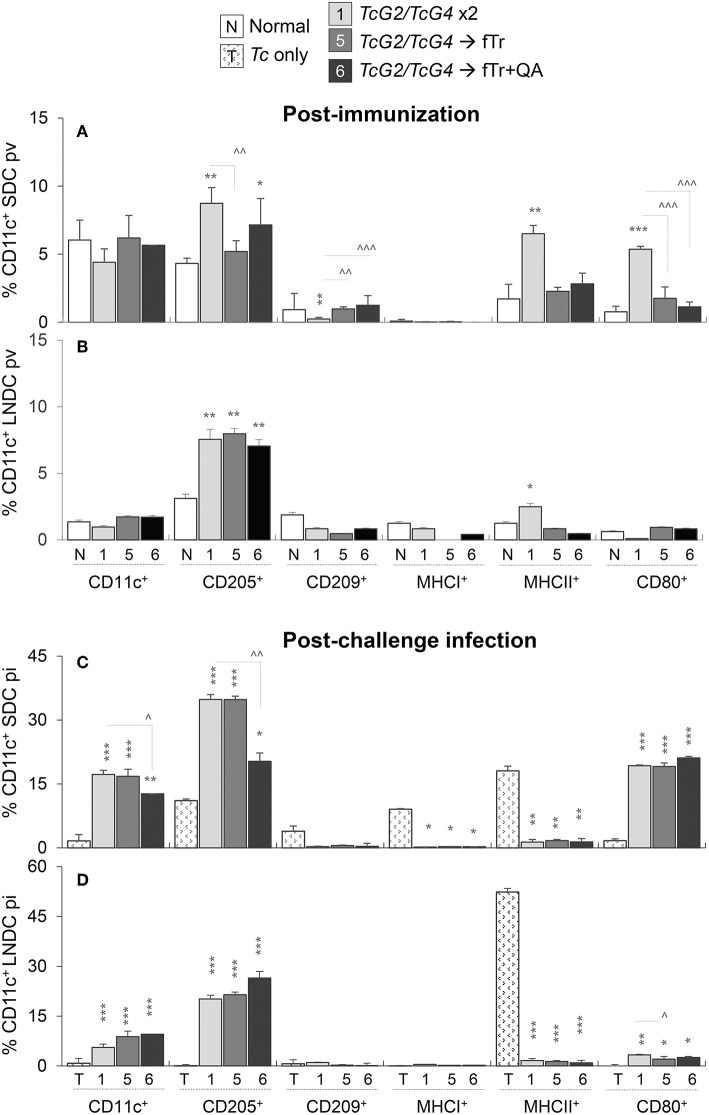
Antigen presenting capacity of dendritic cells (DC) in vaccinated mice (± *T. cruzi*). Mice were vaccinated and challenged with *T*. *cruzi* trypomastigotes (10,000 parasites/mouse), as in Figures 1, 2. Mice were euthanized at 21 days' post-vaccination (pv) or 21 days' post-infection (pi). Single cell suspensions of spleen **(A,C)** and lymph nodes **(B,D)** of vaccinated **(A,B)** and vaccinated/infected **(C,D)** mice were labeled with fluorochrome-conjugated antibodies and analyzed by flow cytometry. Bar graphs show *ex vivo* percentage of Ly6G^lo^CD11c^+^ splenic DC (SDC) and lymph node DC (LNDC) subsets that expressed markers of maturation (CD209^+^), stimulation (CD80^+^), antigen uptake (CD205^+^), and antigen presentation (MHCI^+^, MHCII^+^) in vaccinated and vaccinated/infected mice. SDC and LNDC from non-vaccinated/non-infected (none) and non-vaccinated/infected mice were used as controls. Data are presented as mean ± SD and representative of duplicate observations per sample (*n* = 4 mice per group per experiment). Significance is annotated as * (none vs. vaccinated or infected vs. vaccinated/infected) and ^∧^ (comparison of vaccinated groups) (*,^∧^*p* < 0.05, **,^∧^^∧^*p* < 0.01, and ***,^∧^^∧^^∧^*p* < 0.001).

The overall frequencies of DC (Ly6G^lo^CD11c^+^, spleen: 4.4–6.2%, LN: 0.97–1.7%) as well as of CD209^+^ (maturation marker) and MHCI^+^ (antigen presentation marker) DC were either not changed or slightly decreased in spleen and LN of vaccinated (vs. non-vaccinated/control) mice (Figures 3A,B). However, the splenic frequencies of CD205^+^, MHCII^+^ and CD80^+^ DC, indicating an increase in antigen uptake, processing, and presentation to CD4^+^T cells, were increased by >2-fold in *TcG2/TcG4* DNA vaccinated mice (all, ^*^p < 0.01, Figure 3A). An increase in antigen presenting capacity of LN DC was also evidenced by ~2-fold increase in the frequency of CD205^+^ and MHCII^+^ DC in *TcG2/TcG4* DNA vaccinated mice (^*^p < 0.05, Figure 3B). Importantly, boosting the *TcG2/TcG4* DNA vaccine with fTr (±QA) had none or suppressive effects on the splenic and LN DC activation (Figures 3A,B).

Challenge infection with *T. cruzi* led to > 3-fold increase in the frequency of CD11c^+^ DC and a comparable increase in the frequency of CD205^+^ and CD80^+^ DC in the splenic and LN compartment of mice that were immunized with *TcG2/TcG4* DNA vaccine (compare Figures 3A,B with Figures 3C,D). Boosting with fTr or ftr+QA had non-significant or suppressive effects on *TcG2/TcG4*-induced DC activation post-challenge infection, and frequencies of MHCI^+^ and MHCII^+^ DC were not significantly changed in spleen and LN of vaccinated groups post-infection (Figures 3C,D). Non-vaccinated/infected (vs. normal control) mice exhibited an overall contraction of the splenic and LN levels of Ly6G^lo^CD11c^+^ DC, and the low number of surviving DC primarily exhibited a MHCII^+^ phenotype in non-vaccinated/infected mice (Figures 3C,D). Together, the results presented in Figure 3 suggest that (a) *TcG2/TcG4* DNA vaccine induced DC depot was capable of responding to *Tc* infection by increased proliferation and phenotypic (antigen uptake, processing, and presentation to CD4^+^T cells) activation, and (b) fTr booster was not useful in enhancing the DC response induced by DNA vaccine in mice before or after challenge infection.

### Mφ Activation Profile in Vaccinated Mice (± *Tc*)

**Figure 4:**
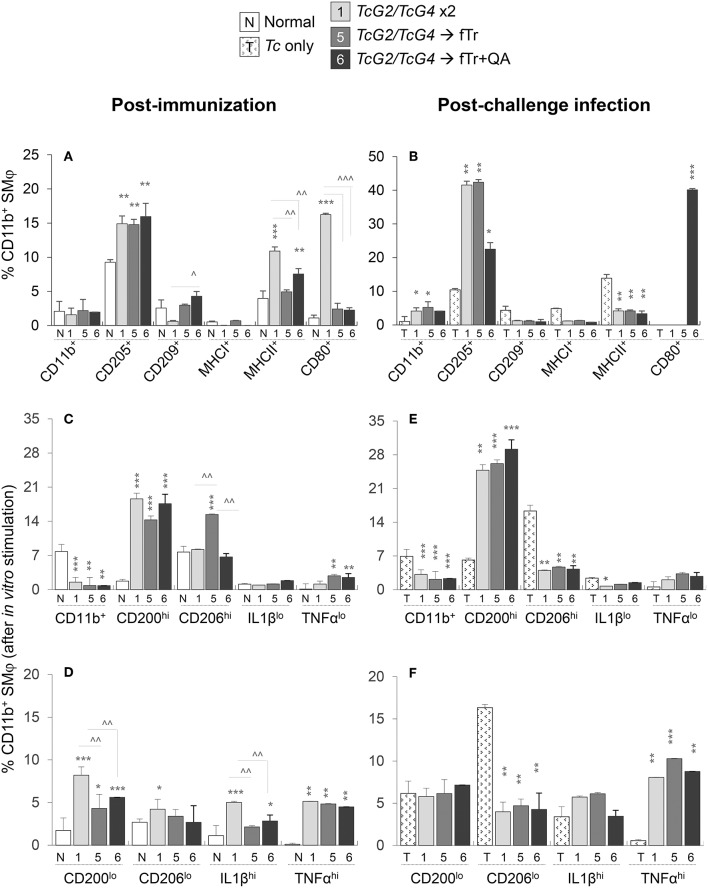
Antigen presenting capacity and functional profile of splenic macrophages (Mφ) in vaccinated mice (± *T. cruzi*). Mice were vaccinated and challenged with *T*. *cruzi* trypomastigotes as in Figures 1, 2. **(A,B)** Splenocytes were labeled with fluorochrome-conjugated antibodies and analyzed by flow cytometry. Bar graphs show *ex vivo* percentages of the splenic Ly6G^lo^CD11b^+^ Mφ that exhibited surface expression of markers of maturation, antigen uptake, and antigen presentation (CD205^+^, CD209^+^, MHCI^+^, MHCII^+^, CD80^+^) in vaccinated **(A)** and vaccinated/infected **(B)** mice. **(C–F)** Splenic cells from vaccinated **(C,D)** and vaccinated/infected **(E,F)** mice were *in vitro* stimulated for 48 h in the presence of soluble *T. cruzi* lysate (TcL), and then labeled with fluorochrome-conjugated antibodies. Shown are the mean percentages of CD11b^+^ Mφ that responded to TcL stimulation with expression of markers of classical/proinflammatory (IL-1β^hi^, TNF-α^hi^) and alternative/immunomodulatory (CD200^hi^, CD206^hi^) phenotype. Splenic cells from non-vaccinated/non-infected and non-vaccinated/infected mice were used as controls. Data (mean ± SD) are representative of two independent experiments (*n* = 3 mice per group per experiment, duplicate observations per mouse), and significance is annotated as *none vs. vaccinated or infected vs. vaccinated/infected, and ^∧^comparison of vaccinated groups (*,^∧^*p* < 0.05, **,^∧^^∧^*p* < 0.01, and ***,^∧^^∧^^∧^*p* < 0.001).

The splenic and LN profile of Mφ are presented in Figure 4 and Figure S2. The overall percentages of Ly6G^lo^Ly6C^+^CD11b^+^ Mφ (range: 1.5–2.2% of total splenocytes) as well as of CD209^+^ and MHCI^+^ Mφs were either not changed or decreased in the splenic compartment of vaccinated mice (vs. non-vaccinated controls, Figure 4A). Mice immunized with *TcG2/TcG4* (gp2) exhibited 60%, 170%, and 1350% increase in the frequencies of CD205^+^, MHCII^+^, and CD80^+^ Mφ, indicating an increase in antigen uptake, processing, and presentation, respectively, to CD4^+^T cells (all, ^**^p < 0.01, Figure 4A), while booster immunization with fTr or fTr+QA (gp5 and gp6) resulted in increased expression of CD205 only (vs. normal controls, Figure 4A). Upon challenge infection, *TcG2/TcG4* vaccinated mice exhibited a further 3-4-fold increase in splenic frequency of Mφ that were primarily CD205^+^ (compare Figures 4A,B). Including fTr or fTr+QA booster dose had either no effects or suppressive effects on CD209^+^, MHCI^+^, and MHCII^+^ Mφ responses after *TcG2/TcG4* vaccination and challenge infection (^∧^*p* < 0.05, Figures 4A,B), and CD80^+^ Mφ were significantly enhanced by fTr+QA booster immunization in challenged mice, suggesting an increase in B7-1 costimulatory signal for T cell activation.

Functional analysis of splenic Mφ after *in vitro* stimulation with *Tc* lysate for 48 h showed 5-9-fold increase in the frequencies of proinflammatory (CD200^lo^, IL-1β^hi^, and TNF-α^hi^) and immunomodulatory (CD200^hi^ and TNF-α^lo^) CD11b^+^ Mφ in *TcG2/TcG4*-vaccinated mice (vs. normal controls, all, ^***^p < 0.05 Figures 4C,D). Splenic Mφ of vaccinated/infected mice responded to *in vitro* stimulation with *Tc* lysate with a further >2-fold increase in the splenic expansion of CD200^hi^ and TNF-α^hi^ Mφ (compare Figures 4C,D with Figures 4E,F). Except for an increase in CD206^hi^CD11b^+^ Mφ population (Figure 4C), inclusion of fTr booster had non-significant effects on *TcG2/TcG4*-induced Mφ activation before or after challenge infection, and co-delivery of QA had an overall suppressive effect on the splenic CD11b^+^ Mφ in vaccinated mice (Figures 4C–F). Non-vaccinated mice exhibited an increase in CD206^lo^ Mφ sub-population post-challenge infection (Figure 4F).

In the LN compartment, non-significant changes or a decline in the percentages of CD205^+^, CD209^+^, MHCI^+^ MHCII^+^, and CD80^+^ Mφ were detected in *TcG2/TcG4*-vaccinated (vs. control) mice (Figure S2A). Inclusion of fTr or fTr+QA booster had an overall suppressive effect on all of the *TcG2/TcG4*-induced sub-populations of LN Mφ, except that fTr+QA booster enhanced the frequency of MHCII^+^CD11b^+^ Mφ in DNA-vaccinated mice (Figure S2A). Further, challenge infection resulted in no increase in the overall percentages of CD11b^+^ LN Mφ in any of the vaccinated groups (Figure S2B). Yet, a substantial phenotypic activation, evidenced by an increase in the frequency of CD205^+^, MHCI^+^, and CD80^+^ Mφ, was noted in LN of *TcG2/TcG4* vaccinated/infected mice (compare Figures S2A,B). Inclusion of fTr (±QA) booster increased the CD205 and CD80 expression and had no significant effect on the CD209 and MHCII expression than that noted in *TcG2/TcG4* immunized mice post-challenge infection (Figure S2B). Non-vaccinated/infected (vs. normal control) mice continued to exhibit a predominance of antigen presenting Mφ (MHCII^+^) in the LN compartment (Figure S2B). Together, the results presented in Figure 4 and Figure S2 suggest that (a) *TcG2/TcG4* DNA vaccine induced Mφ depot was capable of responding to *T. cruzi* infection by increased proliferation, and proinflammatory activation (antigen uptake, processing, and presentation to CD4^+^ T cells; production of IL-1β^+^ and TNF-α cytokines), and (b) fTr based booster immunization had minimal-to-none effects in further enhancing the Mφ response induced by DNA vaccine in mice before or after challenge infection.

### CD4^+^T Cell Profile in Vaccinated Mice (± *Tc*)

**Figure 5:**
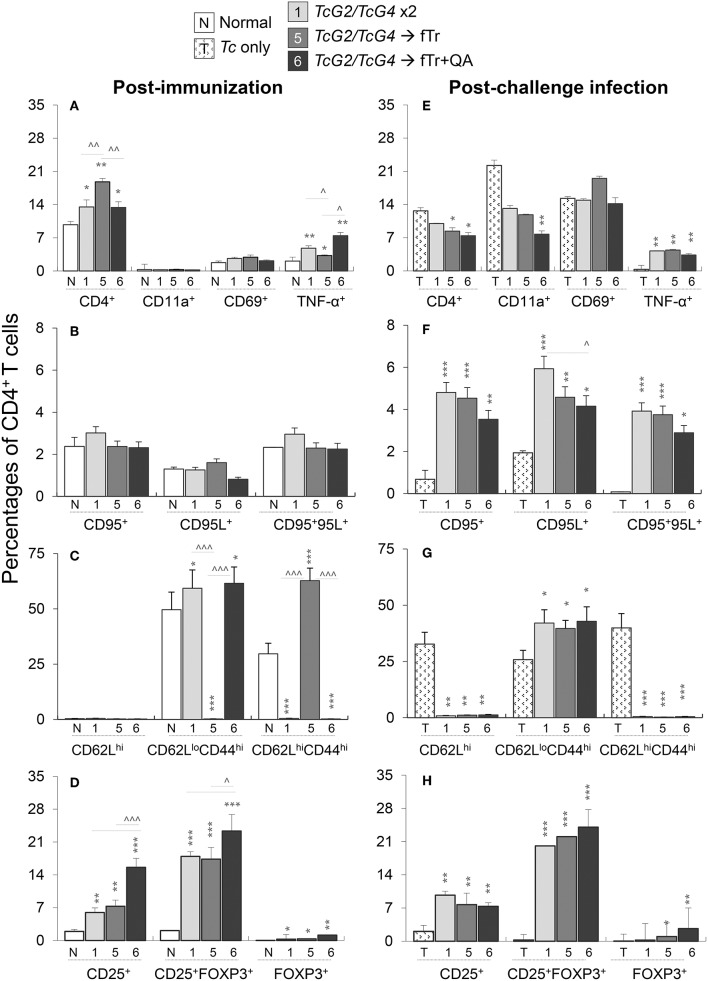
CD4^+^T cell functional profile in vaccinated mice (± *T. cruzi*). Mice were vaccinated and challenged with *T*. *cruzi* trypomastigotes as in Figures 1, 2. Splenocytes from vaccinated **(A–D)** and vaccinated/infected **(E–H)** mice were *in vitro* stimulated for 48 h in the presence of soluble *T. cruzi* lysate, labeled with fluorochrome-conjugated antibodies, and analyzed by flow cytometry. Shown are the percentages of PE^+^CD4^+^ splenic T cells exhibiting the expression of markers of **(A,E)** recruitment (CD11a^+^, CD69^+^) and activation (TNF-α^+^); **(B,F)** cell death (CD95^+^, CD95L^+^); **(C,G)** effector/memory phenotype (CD62L^+^, CD44^+^); and **(D,H)** T regulatory phenotype (CD25^+^ ± FoxP3^+^). Data (mean ± SD) are representative of two independent experiments (*n* = 3 mice per group per experiment, duplicate observations per sample), and significance is annotated as *none vs. vaccinated or infected vs vaccinated/infected, and ^∧^comparison of vaccinated groups (*,^∧^*p* < 0.05, **,^∧^^∧^*p* < 0.01, and ***,^∧^^∧^^∧^*p* < 0.001).

Next, we examined if the *TcG2/TcG4*-induced activation of antigen presenting cells modulate the splenic CD4^+^T cell responses in mice after immunization and challenge infection. For this, splenocytes from vaccinated, vaccinated/infected, and control mice were *in vitro* stimulated with *Tc* lysate, CD3^+^ lymphocytes were gated for CD4^+^T and CD8^+^T cell subsets, and each of the T cell subsets were further analyzed by flow cytometry. Vaccinated (vs. normal control) mice exhibited a significant increase in splenic population of CD4^+^T cells (range: 9.76–18.8% of total) and CD4^+^TNF-α^+^T cells (range: 2.1–7.4% of CD4^+^T cells), respectively (all, ^*^p < 0.05, Figure 5A). Maximal expansion in CD4^+^T cells was noted in mice immunized with *TcG2/TcG4* and boosted with fTr±QA. No significant differences in the expression of other markers of CD4^+^T cell activation (CD69, CD95, CD95L) were noted in any of the vaccinated (vs. normal control) groups (Figure 5B). Interestingly, fTr booster skewed the CD4^+^T cells from effector (CD62L^lo^CD44^hi^) to central memory (CD62L^lo^CD44^hi^) phenotype and addition of QA reversed this effect in vaccinated mice (Figure 5C).

Upon challenge infection, CD4^+^T cells constituted 7.4–12.6% of the total splenocytes, and a large proportion of the CD4^+^T cells exhibited a CD69^+^/CD11a^+^ phenotype (range: 7.7–22%) indicative of T cell recruitment and early activation (Figure 4E). Importantly, CD4^+^T cells in vaccinated/infected (vs. non-vaccinated/infected) mice exhibited >10-fold and 2-6-fold increase in Th1 cytokine (TNF-α) production and Fas/FasL (CD95^+^/CD95L^+^) expression, respectively (^*^p < 0.01, Figures 5E,F), that are required to support CD8^+^T cells in eliminating intracellular infection. Moreover, up to 24% of the naïve CD4^+^T cells also differentiated to induced T regulatory (iTreg) phenotype (CD25^+^FoxP3^+^) in vaccinated mice before and challenge infection (^*^p < 0.01, Figures 5D,H). While adjuvanting with fTr±QA had no effect (data not shown), delivery of fTr±QA as booster dose substantially increased the expansion of CD4^+^T cells and iTreg population when compared to that noted in mice immunized with *TcG2/TcG4* only (all, ^∧^*p* < 0.05, Figures 5A,D). However, fTr±QA booster did not enhance the DNA vaccine induced CD4^+^T cell responses after challenge infection (Figures 5E–H). In non-vaccinated/infected (vs. normal control) mice, the CD4^+^T cells exhibited an increase in the expression of CD11a and CD69, but maintained a predominance of naïve (CD62L^hi^) or central memory (CD62L^hi^CD44^hi^) phenotype (Figures 5E–G). Together, the results presented in Figure 5 suggest that (a) *TcG2/TcG4* DNA vaccine induced CD4^+^T cells acquired the Th1 phenotype evidenced by TNF-α production and expression of markers that orchestrate adaptive immunity to kill intracellular pathogen; (b) a fraction of the vaccine-induced CD4^+^T cells acquired iTreg phenotype to potentially avert the over-reactivity of the immune responses and prevent self-tissue injury; and (c) including fTr booster immunization slightly shifted the CD4^+^T cell response toward iTreg population in vaccinated mice. In comparison, (d) non-vaccinated mice failed to respond to challenge infection with a potent activation of Th1 CD4^+^T cell response.

### CD8^+^T Cell Function in Vaccinated Mice (± *Tc*)

**Figure 6:**
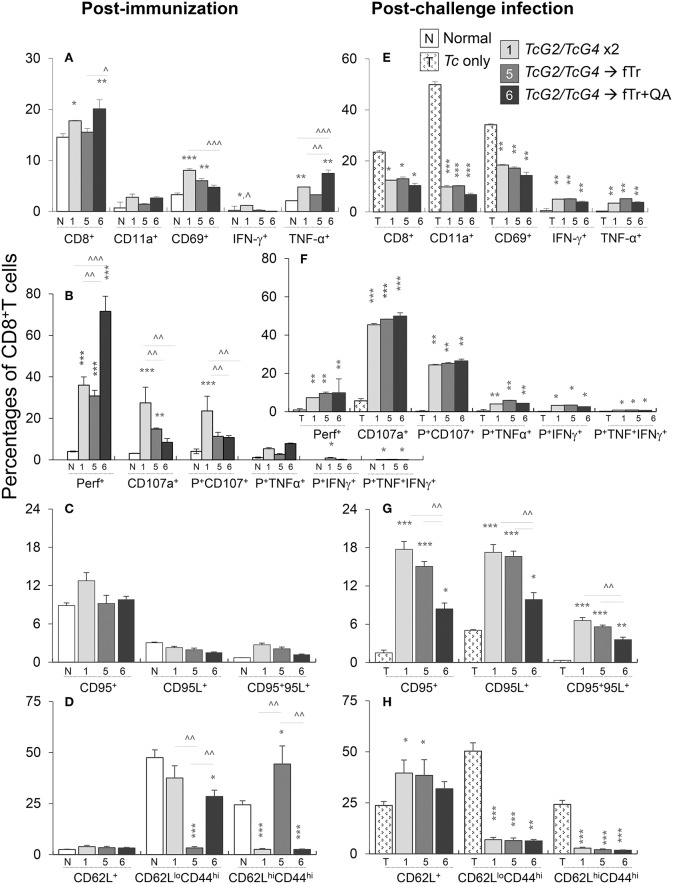
CD8^+^T cell functional profile in vaccinated mice (± *T. cruzi*). Mice were vaccinated and challenged with *T*. *cruzi* as in Figures 1, 2. Splenocytes from vaccinated **(A–D)** and vaccinated/infected **(E–H)** mice were *in vitro* stimulated for 48 h in the presence of *Tc* antigenic lysate, and then labeled with fluorochrome-conjugated antibodies. The FITC^+^CD8^+^T cells were analyzed by flow cytometry. Shown are the mean percentages of **(A,E)** CD8^+^T cells expressing adhesion and migration markers (CD11a^+^, CD69^+^) and type 1 cytokines (IFN-γ and TNF-α) production; **(B,F)** poly-functional CD8^+^T cells that exhibited several markers of cytolytic activity (perforin^+^, CD107a^+^, TNF-α^+^, and IFN-γ^+^); **(C,D)** CD8^+^T cells with expression of cell death markers (CD95^+^, CD95L^+^); and **(C,G)** CD8^+^T cells expressing effector/memory markers (CD44^+^, CD62L^+^). Data (mean ± SD) are representative of two independent experiments (*n* = 3 mice per group per experiment, duplicate observations per sample). Significance is annotated as *none vs. vaccinated or infected vs vaccinated/infected, and ^∧^comparison of vaccinated groups (*,^∧^*p* < 0.05, **,^∧^^∧^*p* < 0.01, and ***,^∧^^∧^^∧^*p* < 0.001).

The splenic CD8^+^T cell response to vaccination and challenge infection is presented in Figure 6. The overall CD8^+^T cell population (range: 17.7–20.1% of total splenocytes) was only slightly increased in vaccinated (vs. control) mice (Figure 6A). Yet, the frequencies of CD8^+^T cells expressing the phenotypic markers of early activation (CD69, 3.29–8.05% of CD8^+^T cells) and cell migration/adhesion (CD11a: 0.69–2.78% of CD8^+^T cells) were increased by 43–144% and 104–302%, respectively, in vaccinated (vs. control) mice (^*^p < 0.05, Figure 6A), the maximal increase being noted in *TcG2/TcG4*-immunized mice. Flow cytometry also revealed that vaccination primed type 1 immunity evidenced by a significant increase in the frequency of IFN-γ^+^ (~1.2% of total, up to 8-fold increase) and TNF-α^+^ (3.2–7.5%, 0.5–2.5-fold increase) CD8^+^T cells with a polyfunctional, cytolytic phenotype (Perforin^+^CD107^+^: 8.4–27.5% of CD8^+^T cells, 1.7–7.8-fold increase; Perforin^+^TNF-α^+^: 2.5–5.4% of CD8^+^T cells, 1.3–3.9-fold increase) when compared to non-vaccinated controls (Figures 6A,B, *p* < 0.05). The maximal increase in CD8^+^T cell frequency, activation, and poly-functional phenotype was noted in mice immunized with *TcG2/TcG4* DNA vaccine only or boosted with fTr+QA. Splenic CD8^+^T cells exhibited no changes in the expression of other markers of activation (CD95/CD95L, Figure 6C). As was noted for CD4^+^T cells, immunization with fTr booster skewed the CD8^+^ T cells from effector (CD62L^lo^CD44^hi^) toward central memory (CD62L^lo^CD44^hi^) phenotype and addition of QA reversed this effect in vaccinated mice (Figure 6D).

Upon challenge infection, the overall splenic percentage of CD8^+^T cells (range: 10.3–13%) contracted in vaccinated mice, yet, CD8^+^T cells in vaccinated mice responded to challenge infection with an increase in the expression of phenotypic markers of T cell recruitment and early activation (CD69: 14.3–18.3% of CD8^+^T cells, CD11a: 6.7–10.2% of CD8^+^T cells, compare Figures 6A,E). Transport of integral membrane proteins' (CD107a and CD107b) to plasma membrane, expression of perforin and granzyme or Fas/FasL (CD95/CD95L), and release of IFN-γ and other cytokines offer effector CD8^+^T cell function through direct killing of the infected cells and/or pleiotropic effects that suppress intracellular pathogen (19). The flow cytometry data showed that vaccinated (vs. non-vaccinated) mice responded to challenge infection with >8-fold increase in the frequency of Th1 cytokine producing (IFN-γ: 3.9–5.1%, TNF-α: 3.5–5.2%) CD8^+^T cells (all, ^*^p < 0.05, Figure 6F). Further, the frequencies of CD8^+^T cells responding to challenge infection with a polyfunctional, cytolytic activation (Perforin^+^CD107^+^: 24–26.5%, Perforin^+^TNF-α^+^: 4.0–5.8%; CD95^+^CD95L^+^: 3.6–6.6%) were significantly increased in vaccinated/infected (vs. non-vaccinated/infected) mice (all, ^*^p < 0.05, Figures 6E–G). The potent increase in CD8^+^T cell frequency, activation, and functional phenotype noted in mice immunized with *TcG2/TcG4* DNA vaccine only was not boosted when fTr±QA were added as adjuvant (data not shown) or as a heterologous booster vaccine (Figure 6). The CD8^+^T cells of non-vaccinated mice responded to challenge infection with an increase in the markers of early activation (CD11a and CD69, Figure 6E), and effector (CD62L^lo^CD44^hi^) or central memory (CD62L^hi^CD44^hi^) phenotype (Figure 6H) but failed to develop polyfunctional cytolytic response (Figures 6E–G). Together, the results presented in Figure 6 suggest that *TcG2/TcG4* vaccine elicited type 1 CD8^+^T cell activation. The vaccine-induced CD8^+^T cells were polyfunctional, and, thus, had a potential to act as cytolytic, effector T cells against *T. cruzi*. The heterologous vaccination with fTr and QA had no clear additive effect in enhancing the *TcG2/TcG4* DNA vaccine induced *Tc*-specific functional CD8^+^ T cell profile.

### Vaccine Efficacy in Controlling Tissue Pathology and *T. cruzi* Infection

**Figure 7:**
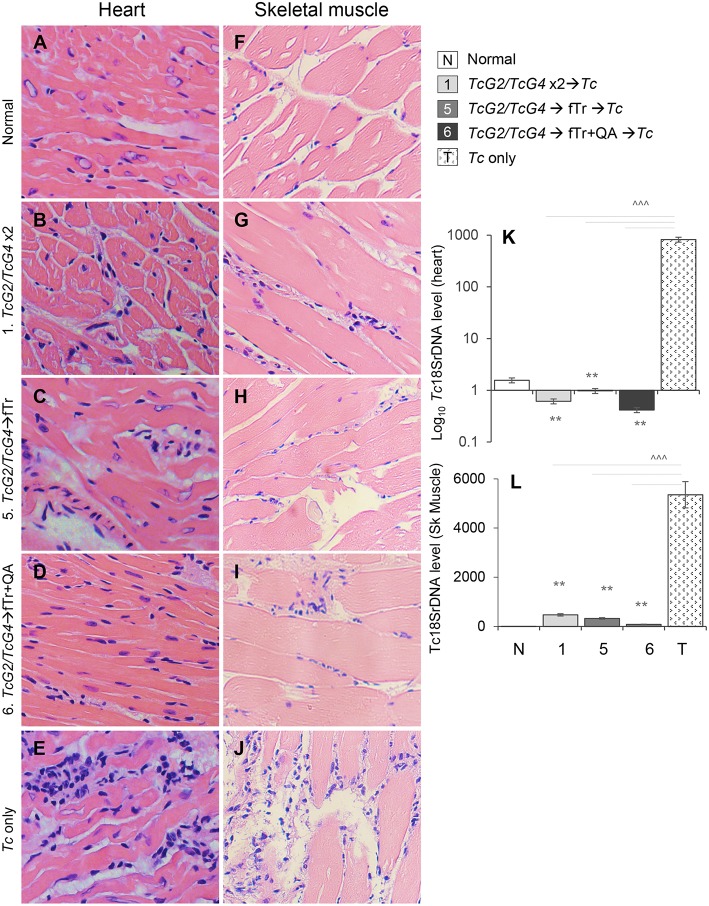
Tissue inflammation and parasite burden in vaccinated mice post-challenge infection. Mice were vaccinated as in Figure 1, challenged with *T. cruzi* at 21 days' post-vaccination and euthanized at 21 days post-infection. **(A–J)** Paraffin-embedded left ventricular heart **(A–E)** and skeletal muscle **(F–J)** tissue sections (5 μM) were examined by hematoxylin/eosin staining (blue: nuclear; pink: muscle/cytoplasm). Shown are representative H&E images of tissue sections from vaccinated/infected and non-vaccinated/infected mice. Non-treated/non-infected mice were used as controls. **(K,L)** Total DNA was isolated from heart **(K)** and skeletal muscle **(L)** tissue sections and submitted to real-time qPCR amplification of *Tc*18SrDNA sequence (normalized to murine *GAPDH*). Data (mean ± SD) are representative of two independent experiments (*n* = 4 mice per group per experiment, triplicate observations per sample). Significance is annotated as *none vs. vaccinated or infected vs vaccinated/infected, and ^∧^comparison of vaccinated groups (*,^∧^*p* < 0.05, **,^∧^^∧^*p* < 0.01, and ***,^∧^^∧^^∧^*p* < 0.001).

Finally, we determined if fTr (±QA) booster immunization enhanced the TcG2/TcG4 vaccine efficacy in controlling inflammatory pathology associated with tissue parasite burden. Histological studies revealed low level of infiltration of inflammatory cells in the heart (Figures 7B–D, histological score: 0–1) and skeletal muscle (Figures 7G–I, histological score: 0–1) of all groups of vaccinated/infected mice. The extent of inflammatory infiltrate in heart and skeletal muscle tissues of non-vaccinated/infected mice (Figures 7E,J, histological score: 2–4) was significantly higher than that detected in tissues of vaccinated/infected mice. Extensive inflammatory foci as well as diffused inflammation, interstitial edema, and loss of tissue integrity were visible in all tissue sections of non-vaccinated/infected mice (Figures 7E,J). In comparison, tissue sections from normal mice showed no inflammatory infiltrate (Figures 7A,F).

The control of tissue parasite burden by immunization was validated by real time qPCR. All vaccinated/infected mice exhibited non-detectable levels of *Tc*18SrDNA in the heart tissue (Figure 7K). The skeletal muscle levels of *Tc*18SrDNA were also barely detectable in vaccinated mice (Figure 7L). A slightly better control of parasite burden in the skeletal muscle was noted in mice vaccinated with *TcG2/TcG4* DNA followed by fTr+QA than in mice given other vaccine compositions (Figure 7L). In comparison, non-vaccinated/infected mice exhibited >10-fold higher levels *Tc*18SrDNA in the myocardial and skeletal muscle tissues post-challenge infection (^∧^*p* < 0.001, Figures 7K,L). Together, these results suggest that *TcG2/TcG4*-based DNA vaccine is highly effective in reducing the acute tissue parasite burden and associated inflammatory pathology, and boosting the TcG2/TcG4-based immunity with fTr (±QA) provides minimal benefits in enhancing the vaccine efficacy against acute *T. cruzi* persistence and tissue injury in mice.

## Discussion

In this study, we have conducted detailed immunological analysis to determine how a homologous DNA-prime/DNA-boost vaccine provides protection from *T. cruzi* infection and examined if the DNA vaccine's efficacy can be complemented with *T. rangeli* as an adjuvant or antigen.

Subunit vaccines are the safest form of vaccine (17, 18). Pre-clinical and clinical trials have demonstrated significant efficacy of subunit vaccines, e.g., THYB-03 (tuberculosis), influvac (influenza), rts,s/AS01 (malaria), gD2gB2-MF59 (HSV2), and RV144 (HIV) (20). In the context of Chagas disease, several candidate antigens (e.g., ASP2, CRP, cruzipain, GP90, GP82, GP56, Tc24, Tc52) have been tested as subunit vaccine, delivered in the form of DNA, recombinant protein, or a host of viral and bacterial expression vectors, in small animal models [reviewed in Padilla et al. (19) and Zak and Aderem (20)]. However, in a majority of the studies, the selected vaccine candidates were chosen based on the preferences or biases of the research teams and investigators. To prevent the investigator bias in vaccine design, we developed a computational/bioinformatic algorithm for screening the *Tc* sequence database for the vaccine candidates (4). We identified 11 potential candidates that were submitted to rigorous analysis for eliciting immunity against *Tc* (5, 8, 21, 24). Eventually, we narrowed down to TcG1, TcG2, and TcG4 candidates that exhibited relevant characteristics as vaccine candidates. These antigens were (1) highly conserved in clinically relevant *Tc* isolates (4), (2) expressed (mRNA/protein) in trypomastigotes and amastigotes (mammalian stages) of *Tc* (4), and (3) recognized by IgGs and type 1 CD8^+^T cells in experimentally or naturally infected mice (5), dogs (6), and humans (7). In a series of studies, we have tested the vaccine potential of TcG1, TcG2, and TcG4 through the use of recombinant eukaryotic plasmid DNA or viral vectors or as a recombinant protein, in eliciting resistance to *Tc* infection (7–11, 16, 18). The homologous DNA-prime/DNA-boost was simplest in composition, and most cost-effective and field-ready, and therefore tested in several compositions (individual candidate antigens, co-delivery of antigenic candidates, different doses and time-intervals, with or without cytokine adjuvants etc.) (4, 5, 23, 25). We found that each of the three candidates elicit antigen-specific IgG and CD8^+^T cell responses and co-delivery of the vaccine candidates elicited additive immune responses (8). The TcG1 candidate was least immunogenic; it stimulated antibody and T cell responses only when it was delivered with IL-12 and GM-CSF adjuvants, and, therefore, it was removed from the later formulations (11). Our published studies discussed above and the present results also suggest that two-dose vaccine delivered at 21-day interval is the most efficacious approach for eliciting anti-parasite protective immune responses.

Our choice of fixed *Tr* as an adjuvant and booster was based on the literature findings that show that *Tr* shares significant antigenic homology with *T. cruzi* (26, 27); *Tr* infects triatomines and mammals but does not induce disease in humans (22, 28), and *Tr* does not circulate in some of the endemic countries (29). Studies in rodents and dogs have shown that exposure to *T. rangeli* or use of heat or chemically attenuated *Tr* elicits antibodies that are reactive against *Tc* antigens, and antibody response was associated with a degree of resistance to infection with *T. cruzi* (30–32). It is also proposed that *Tr* can adjuvant the innate and adaptive immunity against *Tc* infection, though this is not experimentally shown.

Monocytes (Mo), Mφ, and DCs are the mononuclear phagocytic cells that together constitute monocyte phagocyte system (MPS) (33). Mo/Mφ cells elicit the primary immune defense to invading *Tc* by proinflammatory cytokines' expression; and superoxide and nitric oxide (NO) production by the NADPH oxidase (NOX2) and inducible nitric oxide synthase (iNOS) enzymes, respectively (34–36). Along with the cytokines and other mediators they produce, Mφ and DC also serve an important function of antigen presentation to adaptive immune system, and thus strongly influence the magnitude and quality of the vaccine induced protection. Our characterization of DC and Mφ responses in this study showed that *TcG2/TcG4* DNA immunization increased the frequencies of splenic and LN DCs and Mφ expressing markers of antigen uptake, processing, and presentation (e.g., CD205, CD209, MHCII, CD80). The DNA vaccine induced Mφ, despite the immunomodulatory (CD200^hi^) phenotype, exhibited proinflammatory functional activation (TNFα^hi^/IL-1β^hi^) that was further amplified upon *in vitro* stimulation with *Tc* lysate as well as upon challenge infection. The observations that the *TcG2/TcG4-*induced innate immune cells expanded both the antigen presentation and functional profile in response to 2nd stimulation (Figures 3, 4) suggest that DNA vaccine-induced innate immune cells retained memory of *Tc*-specific antigen exposure. Indeed, there is growing appreciation for Mφs' contribution to innate immune memory; a recent study showed Mφ memory to *S. aureus* was tissue specific and transferable between individual animals (37). Also referred as trained immunity, innate immune memory favors the production and release of proinflammatory cytokines (e.g., TNF-α, IL-6, and IL-1β) (38), as we have noted in vaccinated/challenged mice, and it is also defined by metabolic and epigenetic hallmarks. Recent studies have shown that metabolic reprogramming through a shift from oxidative phosphorylation to aerobic glycolysis mediated by Akt/mTOR/HIF-1α pathway is a key mechanism for trained immunity (39); while glycolysis, glutaminolysis, and cholesterol synthesis pathways in Mo/Mφ were suggested to support the epigenetic wiring and induction of improved innate immunity (39, 40). Further studies will be needed to mechanistically demonstrate if *TcG2/TcG4* DNA vaccine promoted the metabolic and epigenetic shifts, and evaluate the longevity of the trained innate immunity and its effects on T cell responses. Yet, our results allow us to surmise that the subunit vaccine design harnessed both the effector response of innate immune cells as well as activation state to enhance adaptive T cell response to the antigens.

Quality and quantity of T cell response, and their cytokine polyfunctionality are critical factors in defining the protective efficacy of vaccine(s) against intracellular pathogens. Immunodominant peptides binding stably to MHC class II on the surface of APCs enhance the recruitment and proliferation of effector CD4^+^ T cells, and secretion of IFN-γ/TNF-α and chemokines by activated APCs and CD4^+^T cells enhance the CD8^+^T cells' accumulation (41). Of a number of immune mediators produced by T cells, IFN-γ and TNF-α play a major role in parasite clearance (10, 12), and production of these cytokines together leads to enhanced killing than either cytokines alone. Additionally, CD8^+^ (and to some degree CD4^+^) T cells mediate cytolytic activity through the release of perforin/granzymes (10). We have noted that DNA immunization of mice enhanced the frequency of Th1 effector CD4^+^T cells and poly-functional, cytotoxic CD8^+^T cells that had the capability to respond to challenge infection with further expansion. The control of tissue parasite burden in immunized mice by >90% suggest that the vaccine induced T cells were highly effective in killing the intracellular pathogen and consequently pathological tissue inflammation was also controlled. CD4^+^ T cells also support immunoglobulin switch and affinity maturation of B cells that enhances the production of neutralizing antibodies; however, we did not notice an increase in quality or quantity of antibody production in vaccinated mice, thus, suggesting that B cell response was not a major feature of the current vaccine design. Another essential feature of the T cells is the type and homing properties of the memory T cell subsets. Whether the current DNA vaccine will provoke long-term/sustained memory T cell response remains to be seen in future studies.

Our results in this study support the existing literature and clearly demonstrate that use of fTr as an adjuvant to subunit DNA vaccine or as an antigen in a booster vaccine dose elicits *Tc*-reactive antibodies that are not elicited by DNA vaccine only. However, fTr did not improve the host outcomes evidenced by the findings that the subunit DNA vaccine delivered by a homologous prime/boost approach was capable of providing >90% control of tissue parasite burden, and inclusion of fTr in a heterologous prime/boost approach did not significantly enhance the efficacy of the DNA vaccine. Further, the inclusion of fTr complicates the efficacy of treatment with anti-parasite drugs or vaccines, as seroconversion is routinely taken as a measure of parasite clearance, and presence of fTr-induced antibodies can hinder the development and efficacy testing of the new drugs and vaccines. Thus, considering the hurdle of facilities, resources and costs associated with the production of fTr, we surmise that inclusion of fTr in subunit vaccine is not needed. These results also provide an explanation for why the first generation of vaccines prepared from the *T. cruzi* parasites (attenuated, killed, fractionated etc.) were not rigorously followed for designing of a commercially viable Chagas vaccine.

With the advancement of CRISPR and other technologies to knockdown genes in trypanosomes, the interest in the development of genetically attenuated live *T. cruzi* vaccine has rekindled (42, 43). The proponents of the live attenuated vaccine believe that such a vaccine will mimic the natural course of infection and provide an appropriate microenvironment of antigen processing and presentation, create a subclinical infection, provide a full-spectrum of antigenic epitopes, and ensure antigen persistence for stimulation of long-lasting immunological responses (44). Yet, other investigators are concerned that an attenuated live vaccine may trigger a full-blown infection and disease in the immunocompromised individuals. Further, a live vaccine, even if protective against one lineage of *T. cruzi*, may not be efficacious in providing protection from infection from other five lineages of *T. cruzi*. Indeed, experimental studies have shown that mice infected with *T. cruzi* and cleared of parasite by treatment with anti-parasite drugs are not protected from re-challenge infection (45). Thus, the concerns in the use of *Tr*- or *Tc*-based attenuated vaccines appear to outweigh the benefits offered by whole organism vaccine in the context of Chagas disease, though this remains to be experimentally proven.

In summary, we have demonstrated that a DNA-prime/DNA-boost vaccine using relatively simple immunogens provides protection from challenge infection. The TcG2/TcG4 DNA vaccine (a) stimulated pro-inflammatory cytokines' production by DC, Mφ, and T cells, and (b) provided antigen uptake, processing, and presentation capacity to cells of the innate immune system that led to the (c) activation of CD4^+^ helper T cells and CD8^+^ polyfunctional, cytolytic T cells. Our data suggest that (d) induction of antibody responses by vaccine is not important for providing protection from infection, and instead an interaction between APCs and adaptive T cell immunity capable of expanding upon challenge infection is sufficient to control *T. cruzi* dissemination. We also conclude that *TcG2/TcG4* vaccine elicits highly effective anti-parasite immunity, and inclusion of attenuated *Tr* (± QA) is not required to improve the DNA vaccine's efficacy against acute *Tc* infection. Future studies should utilize parasites of different lineages, different parasite inoculation size, and monitor all different tissues to ensure that vaccine is fully protective. Our results also encourage the designing of recombinant nano-plasmids that lack antibiotic genes, can incorporate both *TcG2* and *TcG4* genes in one plasmid, and are suitable for large-scale production; and testing of the recombinant nano-plasmids as prophylactic or therapeutic vaccine against *T. cruzi* in multiple hosts.

## Data Availability

All data generated or analyzed during this study are included in this article (and its Supplementary Information Files).

## Ethics Statement

All animal experiments were conducted following the National Institutes of Health guidelines for housing and care of laboratory animals and in accordance with protocols approved by the Institutional Animal Care and Use Committee (protocol number 08-05-029) at The University of Texas Medical Branch at Galveston.

## Author's Note

Chagas cardiomyopathy, caused by a parasitic protozoan *Trypanosoma cruzi*, represents the third greatest tropical disease burden in the world. Based on a decade of research work, we have developed an effective, experimental subunit vaccine composed of two candidate antigens of *T*. *cruzi*. *Trypanosoma rangeli* is non-pathogenic to mammals and shown to elicit cross-reactive anti-*T. cruzi* antibodies. In this study, we investigated if adding fixed *T. rangeli* (fTr) can further enhance the efficacy of the subunit DNA vaccine. We demonstrate that (a) the *TcG2* and *TcG4* DNA vaccine is simplest in design (so far), (b) the candidate antigens elicit highly effective immunity, and (c) inclusion of fTr is not required to improve the DNA vaccine's efficacy against *T. cruzi* infection. Future studies will ensure the efficacy of TcG2/TcG4 based prophylactic and therapeutic immunity against diverse parasite strains, parasite doses, and in multiple hosts.

## Author Contributions

NG provided financial support, conceived the study, and wrote the manuscript. SG, BS-J, and NL designed and performed the experiments, analyzed the data, and confirmed the accuracy of data presentation in the manuscript. JV-C provided *T. rangeli*, and substantially contributed to the conception of the study. All authors have read and approved the manuscript to be published.

### Conflict of Interest Statement

The authors declare that the research was conducted in the absence of any commercial or financial relationships that could be construed as a potential conflict of interest.

## Supplementary Material

Table S1 Splenic and lymph node (LN) cell count post-vaccination.

Table S2 Flow cytometry studies.

Figure S1 Cross-reactivity of antibodies to *T. rangeli* in vaccinated mice. C57BL/6 female mice were immunized with six different compositions of vaccines as described in Materials and Methods. Mice were vaccinated with dose 1 at day 0, dose 2 at day 21, and euthanized at day 42. Vaccines (per dose) were constituted with pCDNA3.*TcG2* and pCDNA3.*TcG4* (25 μg each plasmid DNA, intramuscular), 1 × 10^8^ *T. rangeli* fixed with 0.1 % glutaraldehyde (fTr, subcutaneous), and 5 μg Quil A (QA, subcutaneous). Sera levels of *T. rangeli*-specific IgG **(A)**, IgG1 **(B)**, IgG2A **(C)**, and IgG2b **(D)** antibodies were measured by an ELISA. Sera samples from normal and infected mice were used as controls. Data (mean ± SD) are representative of two independent experiments (*n* = 4 mice per group). Significance is annotated as ^*^none vs. vaccinated or infected vs. vaccinated/infected (^*^*p* < 0.05, ^**^*p* < 0.01, ^***^*p* < 0.001).

Figure S2 Antigen presenting capacity of lymph node macrophages (Mφ) in vaccinated mice (± *T. cruzi*). Mice were vaccinated, infected and euthanized as in Figure S1. Single cell suspensions of lymph node (LN) cells were labeled with fluorochrome-conjugated antibodies, and analyzed by flow cytometry. Bar graphs show *ex vivo* percentages of Ly6G^lo^CD11b^+^ LN Mφ that exhibited surface expression of markers of maturation, antigen uptake, and antigen presentation (CD205^+^, CD209^+^, MHCI^+^, MHCII^+^, CD80^+^) in vaccinated **(A)** and vaccinated/infected **(B)** mice. Lymph node cells from non-vaccinated and non-vaccinated/infected mice were used as controls. Data (mean ± SD) are representative of two independent experiments (*n* = 3 mice per group per experiment, duplicate observations per mouse). Significance is annotated as ^*^none vs. vaccinated or infected vs. vaccinated/infected (^*^*p* < 0.05, ^**^*p* < 0.01, ^***^*p* < 0.001).
