# Supplementary material for: TcG2/TcG4 DNA Vaccine Induces Th1 Immunity Against Acute Trypanosoma cruzi Infection: Adjuvant and Antigenic Effects of Heterologous T. rangeli Booster Immunization
Source: Front Immunol. 2019 Jun 26;10:1456. doi: 10.3389/fimmu.2019.01456 (PMC6606718; doi:10.3389/fimmu.2019.01456)
Supplement: Table S1 — Splenic and lymph node (LN) cell count post-vaccination. [file Table_1.DOCX]

**S1 Table: Splenic and lymph node (LN) cell count post-vaccination**

| **Groups** | **Splenocytes (1 x 10^6^)** | **LN cells (1 x 10^6^)** |
| --- | --- | --- |
| N. Normal control | 80 ± 22 | 144 ± 36 |
| 1. *TcG2/TcG4* x2 | 193 ± 52 | 148 ± 32 |
| 2. *TcG2/TcG4*+fTr x2 | 166 ± 35 | 192 ± 26 |
| 3. *TcG2/TcG4*+QA x2 | 142 ± 22 | 144 ± 32 |
| 4. *TcG2/TcG4*+fTr+QA x2 | 130 ± 25 | 150 ± 30 |
| 5. *TcG2/TcG4* 🡪 fTr | 176± 35 | 200 ± 20 |
| 6. *TcG2/TcG4* 🡪 fTr+QA | 170 ± 32 | 220 ± 18 |
| C57BL/6 female mice were immunized with six different compositions of vaccines as described in Materials and Methods. Mice were immunized with dose 1 at day 0 and dose 2 at day 21. Vaccines (per dose) were constituted with pCDNA3.*TcG2* and pCDNA3.*TcG4* (*TcG2/TcG4*, 25 μg each plasmid DNA in 100 μl PBS, intramuscular) and 1X10^8^ *T. rangeli* fixed with 0.1% glutaraldehyde (fTr, subcutaneous) in 100 μl PBS with or without 5 µg Quil A (QA, subcutaneous)*.* Splenic and LN cell counts were determined at 21 days’ post vaccination (pv) by light microscopic evaluation of single cell suspensions (n = 4 per group per experiment). **Note:** No significant differences were noted in splenic cell count in any of the groups post-challenge infection with *T. cruzi*. | | |
