# Supplementary material for: TcG2/TcG4 DNA Vaccine Induces Th1 Immunity Against Acute Trypanosoma cruzi Infection: Adjuvant and Antigenic Effects of Heterologous T. rangeli Booster Immunization
Source: Front Immunol. 2019 Jun 26;10:1456. doi: 10.3389/fimmu.2019.01456 (PMC6606718; doi:10.3389/fimmu.2019.01456)
Supplement: Table S2 — Flow cytometry studies. [file Table_2.DOCX]

| **S2 Table: Flow cytometry studies** | | | | | |
| --- | --- | --- | --- | --- | --- |
| **Marker** | **Color / Format** | **Ex / Em(nm)** | **Antibody cat#** | **Source** |  |
| **General exclusion markers** | | | | | |
| CD3 epsilon | Alexa Fluor 488 | 488 / 530/30 | Hamster α m / IgG (500A2) | Thermo Fisher (HM3420) |  |
| CD45R/B220(B cell) | DyLight 680 | 640 / 730/44 | Rat α m / IgG2a (RM0063-9F14) | Novus (NBP2-12168FR) |  |
| CD19 (B cell) | FITC | 490 / 530/30 | Rat α m / IgG2a, κ (eBio1D3) | Thermo Fisher (11-0193-82) |  |
| Ly-6G (Granulocytes) | Brilliant Violet 650 | 405 / 650/30 | Rat α m / IgG2a, κ (1A8) | BD Biosc (740554) |  |
| Ly6G (Granulocytes) | V450 | 405 / 450/50 | Rat α m / IgG2a, κ (1A8) | BD Biosc (560603) |  |
| **Antigen presentation and processing** | |  |  |  |  |
| CD11b (Mϕs) | Brilliant violet 605 | 405 / 610/20 | Rat α m / IgG2a κ (M1/70) | BD Biosc (563015) |  |
| CD11c (DCs) | Brilliant Violet 785 | 405 / 780/60 | Hamster α m / IgG (N418) | Biolegend (117336) |  |
| CD205 (Ag uptake) | PerCP e-fluor | 488 / 710/50 | Rat α m / IgG2a, κ (205yekta) | Thermo Fisher (46-2051-80) |  |
| CD209 (Maturation) | FITC | 490 / 530/30 | Rat α m / IgG2a, κ (LWC06) | Thermo Fisher (1-2092-82) |  |
| MHC I (Ag expression) | DyLight 350 | 355 / 450/50 | Mouse α m / IgG2a κ (14-4-4S) | Novus (NBPI-43736UV) |  |
| MHC II (Ag expression) | PE | 561 / 582/14 | Mouse α m / IgG1 κ (OX-6) | BD Biosc (554929) |  |
| CD80 (Co-stimulatory) | APC | 594 / 670/30 | Rat α m / IgG2a κ (1G10) | Thermo Fisher (A14724) |  |
| CD 206 (Class II) | APC5 | 594 / 670/30 | Rat α m / IgG2b (MR6F3) | Thermo Fisher (17-2061-82) |  |
| CD200 (Class II) | PE | 561 / 582/14 | Rat α m / IgG2a κ (OX-90) | Thermo Fisher (12-5200-82) |  |
| TNF-α (Class I) | FITC | 490 / 530/30 | Rat α m / IgG1κ (MP6-XT22) | Thermo Fisher (11-7321-82) |  |
| IL-1β (Class I) | PE-Cy7 | 496 / 785 | Rat α m / IgG1 κ (NJTEN3) | Thermo Fisher (25-7114-80) |  |
| **T cell activation and function** | |  |  |  |  |
| CD4 (T cell) | V450 | 405 / 450/50 | Rat α m / IgG2a κ (RM4-5) | BD Biosc (560468) |  |
| CD8 (T cell) | V500 | 405 / 525/50 | Rat α m / IgG2a κ (53-6.7) | BD Biosc (560776) |  |
| TNF-α cytokine | PerCP-Cy5.5 | 488 710/50 | Rat α m / IgG1 κ (MP6-XT22) | BD Biosc (560659) |  |
| IFN-γ cytokine | APC | 594 / 670/30 | Rat α m / IgG1 κ (XMG1.2) | Thermo Fisher (17-7311-82) |  |
| Perforin (Cytotoxicity) | FITC | 490 / 530/30 | Rat α m / IgG2a κ (eBioOMAK) | Thermo Fisher (11-9392-82) |  |
| CD107a (Degranulation) | PE | 561 / 582/14 | Rat α m / IgG2a κ (ID4B) | BD Biosc (558661) |  |
| CD11a (LFA-1) | PE-Cy7 | 496 / 785 | Rat α m / IgG2a κ (2D7) | BD Biosc (558191) |  |
| CD69 (T cell activation) | PE | 561 / 582/14 | Hamster α mouse (HI.2F3) | BD Biosc (553237) |  |
| CD62L (Memory) | PerCP-Cy5.5 | 482 / 710/50 | Rat α m/ IgG2a κ (MEL-14) | Thermo Fisher (45-0621-82) |  |
| CD44 (Memory) | APC | 594 / 670/30 | Rat α m / IgG2b κ (IM7) | Thermo Fisher Sc (17-0441-82) |  |
| CD25 (T_reg_ activation) | PE | 561 / 582/14 | Rat α m / IgG1 λ (PC61.5) | Thermo Fisher (25-0251-82) |  |
| FoxP3 (T_reg_ activation) | PerCP-Cy5.5 | 482 / 710/50 | Rat α m / IgG2a κ (R16-715) | BD Biosc (563902) |  |
| CD95 (Cell death receptor) | PE-Cy7 | 496 / 785 | Hamster α m / IgG2.λ2 (Jo-2) | BD Biosc (557653) |  |
| CD95L/CD178  (cell death ligand) | PE | 561 / 582/14 | Hamster α m / IgG1 κ (MFL3) | BD Biosc (555293) |  |

Fluorescence conjugated antibodies that were used to evaluate the effect of vaccine and challenge infection with *T. cruzi* on the splenic and lymph node immune responses in mice are listed.
