# Supplementary figures and images for: TcG2/TcG4 DNA Vaccine Induces Th1 Immunity Against Acute Trypanosoma cruzi Infection: Adjuvant and Antigenic Effects of Heterologous T. rangeli Booster Immunization
Source: Front Immunol. 2019 Jun 26;10:1456. doi: 10.3389/fimmu.2019.01456 (PMC6606718; doi:10.3389/fimmu.2019.01456)

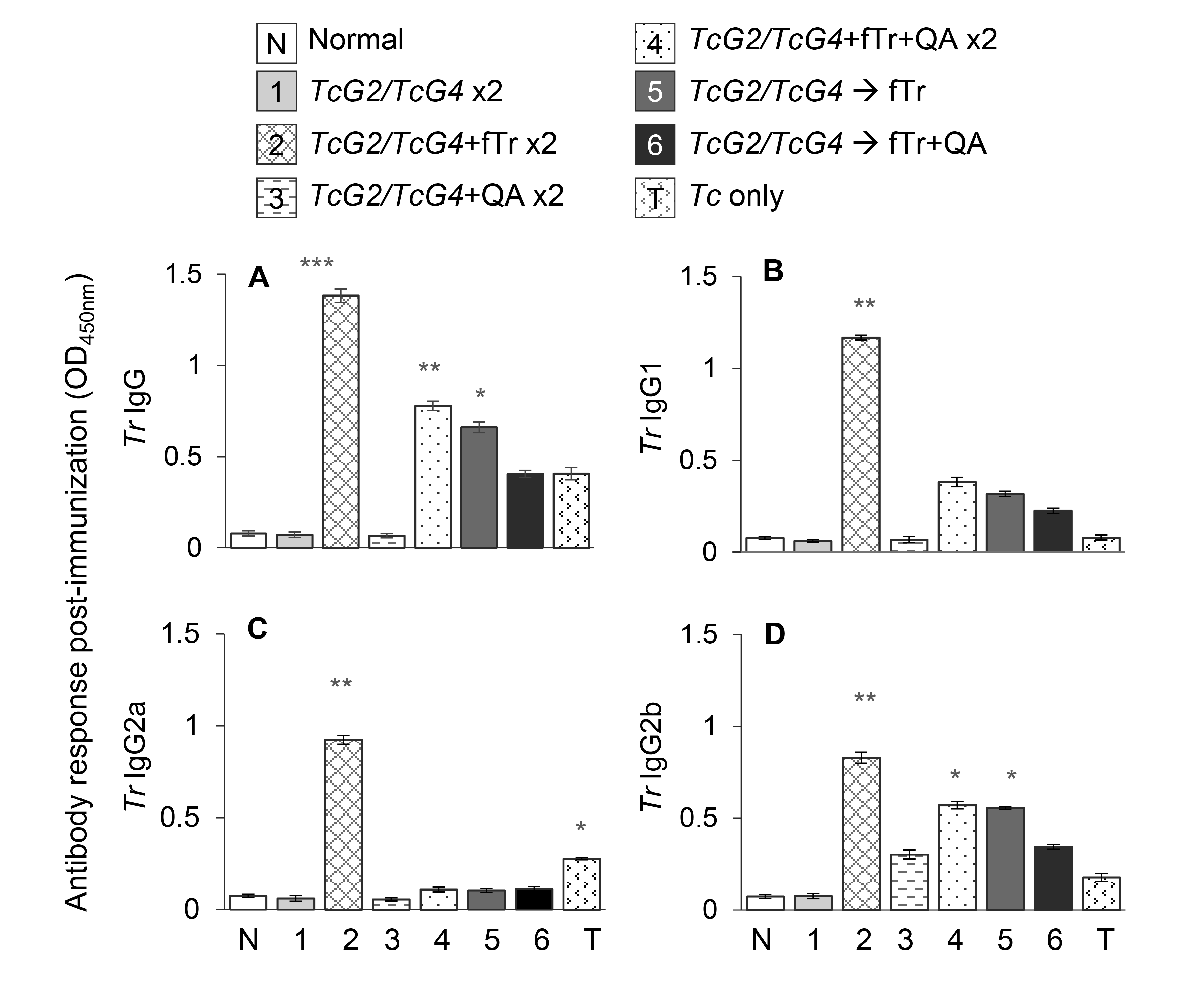

Supplement: Figure S1 — Cross-reactivity of antibodies to T. rangeli in vaccinated mice. C57BL/6 female mice were immunized with six different compositions of vaccines as described in Materials and Methods. Mice were vaccinated with dose 1 at day 0, dose 2 at day 21, and euthanized at day 42. Vaccines (per dose) were constituted with pCDNA3.TcG2 and pCDNA3.TcG4 (25 μg each plasmid DNA, intramuscular), 1 × 10^8^ T. rangeli fixed with 0.1 % glutaraldehyde (fTr, subcutaneous), and 5 μg Quil A (QA, subcutaneous). Sera levels of T. rangeli-specific IgG (A), IgG1 (B), IgG2A (C), and IgG2b (D) antibodies were measured by an ELISA. Sera samples from normal and infected mice were used as controls. Data (mean ± SD) are representative of two independent experiments (n = 4 mice per group). Significance is annotated as *none vs. vaccinated or infected vs. vaccinated/infected (*p < 0.05, **p < 0.01, ***p < 0.001). [file Image_1.TIF]

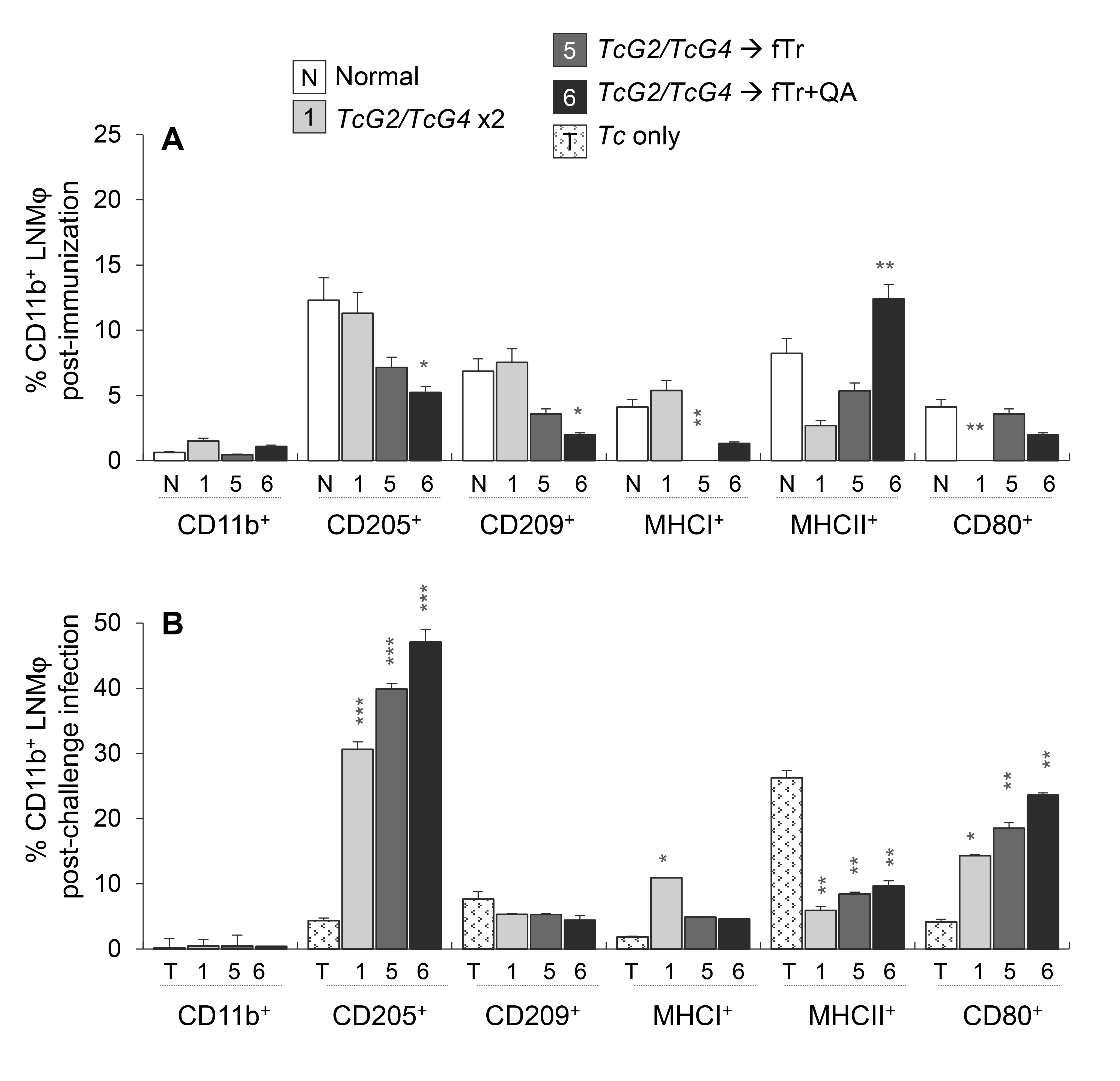

Supplement: Figure S2 — Antigen presenting capacity of lymph node macrophages (Mφ) in vaccinated mice (± T. cruzi). Mice were vaccinated, infected and euthanized as in Figure S1. Single cell suspensions of lymph node (LN) cells were labeled with fluorochrome-conjugated antibodies, and analyzed by flow cytometry. Bar graphs show ex vivo percentages of Ly6GloCD11b+ LN Mφ that exhibited surface expression of markers of maturation, antigen uptake, and antigen presentation (CD205+, CD209+, MHCI+, MHCII+, CD80+) in vaccinated (A) and vaccinated/infected (B) mice. Lymph node cells from non-vaccinated and non-vaccinated/infected mice were used as controls. Data (mean ± SD) are representative of two independent experiments (n = 3 mice per group per experiment, duplicate observations per mouse). Significance is annotated as *none vs. vaccinated or infected vs. vaccinated/infected (*p < 0.05, **p < 0.01, ***p < 0.001). [file Image_2.TIF]
